# Case of Precocious Development of the Sexual System in a Female Child

**Published:** 1853-12

**Authors:** Charles Wilson

**Affiliations:** New Berlin, Union Co., Pa.


*Case of Precocious Development of the Sexual System in a Female Child.* By CHARLES WILSON, M. D., of New Berlin, Union Co., Pa.

In Nov. 1850, while residing at Selin's Grove, in this county, I was called several miles into the country, at night, to see a case of croup. After the little sufferer was relieved and I was about to depart, the mother of the child requested me to prescribe for a daughter who had been in ill health for several weeks, on account of a suppression of the menses. I replied that as it depended altogether upon the state or condition of health her daughter was in, it would be necessary for me to see her before I could prescribe an appropriate remedy. She then stated that her daughter was not yet six years old, and was so bashful that I could get nothing out of her, nor would she remain in my presence. On my naturally expressing much surprise at what she related, and in answer to my earnest questions, the mother gave me the following history of her daughter's case:

Mary Ann G—— was born in March, 1845. There was nothing remarkable observed in the child at birth, but the unusual size of the mammæ, which were "as large as hen's eggs;" they increased rapidly, and at the fifth month had attained the size of a girl at puberty. The mother at this time noticed the child's diaper stained with blood, and upon examination, ascertained that it proceeded from the genitals. This discharge continued two days. In five months subsequently,—the tenth month of her age,—this discharge reappeared and continued for three days. After this it came on every three months, until she was four years old, when it did not appear at the accustomed time. The child fell into bad health and had the usual ailings of females laboring under a suppression. After using various remedies, she was again regular at the seventh month from the first period, when she immediately regained her former good health. Her menses, however, have since then appeared only every seven months; they continue, and have done so for several years, five days. Now, however, her time to be regulated has passed for some weeks without a show, and she again suffers bad health, for which I am consulted.

On expressing an anxious desire to the father to see and examine the child, he gratified me by leading the way to her chamber, where she lay on her bed asleep, and completely exposed her. She was about the ordinary stature of children of her age, but unusually heavy set and fat. Her breasts were about the size of a *well developed* adult virgin's, of which her father said she used to feel very proud, and take boastful airs on herself with her little playmates, but latterly has become shy and avoids any notice of or allusion to them by others. The pudendum was thinly covered with black hair, and altogether she presented the appearance of a girl after the age of puberty.

In answer to my interrogatory, whether she ever manifested any amorous predilections for the other sex, the father replied that he had observed none.

I prescribed for this anomalous little creature, but heard nothing more of her, as her parents shortly afterwards removed to another and more distant residence.

